# Morbidity associated with patent ductus arteriosus in preterm newborns: a retrospective case-control study

**DOI:** 10.1186/s13052-021-00956-2

**Published:** 2021-01-14

**Authors:** Gianluca Terrin, Maria Di Chiara, Giovanni Boscarino, Valentina Metrangolo, Francesca Faccioli, Elisa Onestà, Antonella Giancotti, Violante Di Donato, Viviana Cardilli, Mario De Curtis

**Affiliations:** Department of Maternal and Child Health, University of Rome La Sapienza, Rome, Italy

**Keywords:** Bronchopulmonary dysplasia (BPD), Necrotizing enterocolitis (NEC), Retinopathy of prematurity (ROP), Intraventricular hemorrhage (IVH), Mortality, Survival, Ibuprofen, Paracetamol

## Abstract

**Introduction:**

Association between persistency of a patent ductus arteriosus (PDA) and morbidity in preterm newborns is still controversial. We aimed to investigate the relation between PDA and morbidity in a large retrospective study.

**Methods:**

A case-control study including neonates consecutively admitted to the Neonatal Intensive Care Unit (NICU), with gestational age (GA) < 32 weeks or body birth weight (BW) < 1500 g, over a 5-year period. Newborns were divided into Cases and Controls, according with the presence or absence of a hemodynamically significant PDA (hs-PDA).

**Results:**

We enrolled 85 Cases and 193 Controls. Subjects with hs-PDA had significantly (*p* < 0.001) lower GA (26.7 w, 95%CI 27.1–28.0 vs. 30.1 w, 95%CI 29.7–30.4), BW (1024 g, 95% CI 952–1097 vs. 1310 g 95%CI 1263–1358) and an increased morbidity (60.0% vs. 18.7%). In a sub-group of extremely preterm newborns (GA ≤ 28 weeks and BW ≤ 1000 g), the rate of bronchopulmonary dysplasia (BPD) was significantly increased in Cases (31.7%) compared with Controls (5.9%, *p* = 0.033). Multivariate analysis showed that morbidity significantly depended on hs-PDA, GA and BW, and that, in extremely preterms, the hs-PDA represented an independent risk factor for BPD.

**Conclusions:**

Occurrence of the main morbidities of prematurity depended by hs-PDA, in association with GA, BW, and use of prenatal steroids. In extremely premature babies, hs-PDA is a risk factor for BPD, one of the most important morbidity of prematurity, independently by other confounding variables.

**Supplementary Information:**

The online version contains supplementary material available at 10.1186/s13052-021-00956-2.

## Introduction

The ductus arteriosus is a vascular structure that connects the proximal descending aorta to the roof of the main pulmonary artery near the origin of the left branch pulmonary artery [1]. The ductus arteriosus normally constricts after birth and becomes functionally closed by the first days of life [2]. Persistency of a patent ductus arteriosus (PDA) is a common issue in extremely low birth infants. Particularly, in those extremely low birth weight (ELBW) newborns, the duct fails to close leading to a left heart volume overload and systemic hypoperfusion [2]. Clinical consequences of this hemodynamic condition are still controversial. It is worth of mentioning that the effects of a hemodynamically significant PDA (hs-PDA) on neonatal morbidities remain widely unproven. If a body of evidence suggests an increase in the morbidity associated with PDA, recent randomized controlled studies and metanalysis have failed to demonstrate a causal relationship [2–5]. However, an association between PDA and morbidity in ELBW newborns cannot be definitive excluded from current literature review. In addition, in many studies criteria for diagnosis hs-PDA were not clearly described.

The purpose of this study was to investigate the association between the presence of PDA and the occurrence of morbidities in very low birth weight (VLBW) newborns, focusing on newborns with gestational age (GA) ≤ 28 weeks and birth weight (BW) ≤ 1000 g.

## Methods

### Study design and population

This is a case-control study including all neonates consecutively admitted to the Neonatal Intensive Care Unit (NICU) of Policlinico Umberto I Hospital, La Sapienza University of Rome, from April 2015 to March 2020, with GA < 32 weeks or body BW < 1500 g. We excluded newborns with incomplete clinical data, major congenital malformations, inborn errors of metabolism, intestinal and extra-intestinal congenital diseases, familiar history of allergy, use of pre-or probiotics, transfer to other hospital or death within the first 72 h of life [6–11].

The enrolled subjects were divided into 2 Groups according to the presence (Cases) or absence (Controls) of a hs-PDA, confirmed by echocardiographic evaluation. We defined PDA, as hemodynamically significant, when we observed at least one of the following criteria, other than PDA diameter at its narrowest part ≥1.5 mm: 1) unrestrictive pulsatile transductal flow; 2) left atrial-to-aortic root ratio ≥ 1.5; 3) absent diastolic flow in descending aorta; 4) abnormal diastolic flow in middle cerebral artery [5, 12–14]. Echocardiography (ECHO) was routinely performed by cardiologists trained in neonatal functional ECHO between 24 and 72 h after birth, in all premature infants admitted in NICU. A second echocardiographic evaluation was performed at 7 days of life. The ECHO evaluation was performed by two cardiologists with high training, unaware of the study aims. Each measurement was confirmed after an agreement between the two cardiologists. They performed each measurement three times and then reported the mean value in a specific data form.

Newborns were classified as Cases when affected by hs-PDA, confirmed by echocardiographic evaluations performed during the first week of life. We classified as Controls all newborns without hs-PDA during hospital stay. Pediatric cardiologists performed ultrasound studies and the treatment was eventually decided after an agreement between cardiologists and neonatologists on duty.

To conduct this research, we compared two historical Groups of newborns using data collected during a study previously approved by Ethical Committee (n°5089). We obtained a written informed consent from all parents of enrolled newborns. This study was conducted in conformity with World Medical Association Declaration of Helsinki for medical research involving human subjects.

### Outcomes

We considered as primary outcome the rate of newborns with at least one of the following morbidities, before discharge from the NICU: prolonged mechanical ventilation (more than 7 days), bronchopulmonary dysplasia (BPD), intraventricular haemorrhage (IVH) stage≥2, necrotizing enterocolitis (NEC) Bell-Stage ≥2, retinopathy of prematurity (ROP) stage ≥3, hypotension [1, 2, 15–17]. We considered as secondary outcomes the mortality and the duration of hospital stay.

### Data collection

Investigators who were not involved in the enrollment phases, recorded clinical data using a structured data form, from birth to discharge, transfer to another hospital or death of the patient. We collected data about gender, GA, BW, twin pregnancies, type of delivery, use of antenatal steroids, pregnancy-induced hypertension, alteration of Doppler velocimetry on uterine arteries, pH on cord blood and Apgar score at 5 min.

Diagnosis of the main neonatal morbidities were performed according to the standard criteria, by physicians unaware of the study design and aims, as previously described [18–20]. Nutritional management was performed as previously described [18, 21–23]. We collected data on morbidity and duration of hospital stay in a separate and codified data form.

### Statistics

Statistical analysis was performed per protocol, using Statistical Package for Social Science software for Microsoft Windows (SPSS Inc., Chicago, IL), and version 25.0. We checked for normality using Shapiro-Wilk test. The mean and 95% confidence interval (CI) summarised continuous variables. We compared the Groups using chi-square test for categorical variable and t-test or Mann-Whitney for paired and unpaired variables.

We performed a sensitivity analysis, including a sub-group of patients with GA ≤ 28 weeks (Sub-group A) and BW ≤ 1000 g (Sub-group B). We performed a binary logistic regression analysis to evaluate whether gender (female or male), GA (< 29 or ≥ 29 weeks of PMA) or BW (≤ 1000 or > 1000 g), use of prenatal steroids, pH at birth (< 7.1 or ≥ 7.1), and Group (Case or Control) or Sub-Group assignment (A or B) influenced primary outcome. We performed linear regression analysis to evaluate if gender, GA or BW and antenatal steroids or pregnancy-induced hypertension influenced duration of hospitalization. The level of significance for all statistical tests was 2-sided (*p* < 0.05).

## Results

We considered eligible for the study 343 newborns. We excluded 65 neonates because of congenital malformations, congenital infections, maternal autoimmune diseases (*n* = 29), early transfer to other hospital (*n* = 21) or death (*n* = 15). Thus, we included 278 infants, 85 as Cases (30.6%) and 193 as Controls (69.4%).

The main clinical characteristics of participating Cases and Controls subjects were summarized in Table 1. We observed that infants with hs-PDA had lower GA, BW, Apgar scores at 5 min, rate of administration of antenatal steroids and pH on cord blood.

**Table 1:** Clinical characteristics of study population

	***Overall***		***Gestational Age ≤ 28 weeks and Birth Weight*** ***≤ 1000 g***	
	Case Group *(n = 85)*	Control Group *(n = 193)*	Case Group *(n = 41)*	Control Group *(n = 18)*
Female sex, *N. (%)*	41 (48.2)	87 (45.1)	22 (53.7)	8 (44.4)
Gestational Age, *weeks*	27.6 (27.1 to 28.0) *	30.1(29.7 to 30.4)	25.9 (25.4 to 26.4)	26.0 (25.1 to 26.9)
Birth weight, *g*	1024 (952 to 1097) *	1310 (1263 to 1358)	754.1 (709.6 to 798.6)	769.5 (681.2 to 875.8)
Twins, *N. (%)*	23 (27.1)	54 (28.0)	8 (19.5)	5 (27.8)
Cesarean Section, *N. (%)*	69 (82.1)	168 (88.4)	27 (65.8)	13 (72.2)
Antenatal steroids ^a^, *N. (%)*	49 (59.8) *	139 (73.5)	28 (68.3)	12 (66.7)
Pregnancy-induced hypertension, *N. (%)*	20 (26.3)	46 (24.1)	14 (34.1) *	1 (5.5)
Alteration of doppler velocimetry of uterine arteries, *N. (%)*	14 (19.4)	35 (18.5)	3 (7.3)	3 (16.7)
pH on cord blood	7.2 (7.2 to 7.3) *	7.3 (7.2 to 7.3)	7.2 (7.2 to 7.3)	7.2 (7.2 to 7.3)
5-min Apgar score	7 (6 to 7) *	8 (7 to 8)	6.6 (6.1 to 7.1)	7.1 (6.5 to 7.6)

Data were expressed as mean (95% CI), when not specified

(^a^) Intramuscular steroid cycle in two doses of 12 mg over a 24-h period; * vs Control Group *p* < 0.05.

The overall morbidity was significantly increased in Cases compared with Controls (Table 2). The risk of prolonged mechanical ventilation, BPD, IVH, ROP and hypotension was increased in Cases compared with Controls (Table 2). The rate of mortality was increased in Cases (10.6%) compared with Controls (3.6%, *p =* 0.025). Mean duration of hospital stay was increased in newborns with hs-PDA (80.4 days, 95%CI 69.4 to 91.4) compared with Controls (54.9 days, 95%CI 50.9 to 58.9, *p <* 0.001).

**Table 2:** Morbidity associated with hemodynamic significant patent ductus arteriosus

	***Overall***		***Gestational Age ≤ 28 weeks and Birth Weight*** ***≤ 1000 g***	
	Case Group *(n = 85)*	Control Group *(n = 193)*	Case Group *(n = 41)*	Control Group *(n = 18)*
Prolonged Mechanical Ventilation	19 (22.4) *	11 (5.7)	17 (41.5)	3 (16.7)
Bronchopulmonary Dysplasia	13 (15.3) *	6 (3.1)	13 (31.7) *	1 (5.9)
Intraventricular Hemorrhage	17 (20.0) *	8 (4.1)	12 (29.3)	4 (22.2)
Necrotizing Enterocolitis	1 (1.2)	5 (2.6)	1 (2.4)	1 (5.6)
Retinopathy of prematurity	18 (21.2) *	5 (2.6)	14 (34.1)	2 (11.1)
Hypotension	27 (31.8) *	11 (5.7)	19 (46.3)	6 (33.3)
Overall Morbidity^a^	51 (60.0) *	36 (18.7)	34 (82.9)	12 (66.7)

Data were expressed as No. (%)

(^a^) BPD or Prolonged mechanical ventilation or IVH or NEC or ROP or Hypothension; * vs Control Group *p* < 0.05

Multivariate analysis showed that morbidity was significantly related to the occurrence of a hs-PDA, in both models including GA and BW, respectively (Table 3). Mortality was not influenced by the presence of a hs-PDA but depended only on GA and BW (Table 3). Multivariate linear regression analysis showed that duration of hospital stay was influenced by the occurrence of hs-PDA along with GA ≤ 28 weeks, BW ≤ 1000 g and the administration of antenatal steroids, in all enrolled newborns (Table S1).

**Table 3:** Multivariate analysis evaluating confounding variables on morbidity and mortality

*Dependent variables*	Covariates (Model 1)				Covariates (Model 2)			
	hs-PDA	Gender	GA ≤ 28 weeks	Antenatal steroidsa	hs-PDA	Gender	BW ≤ 1000 g	Antenatal steroids^a^
Prolonged Mechanical Ventilation								
*OR (95%CI)*	2.567 (1.063 to 6.202) *	1.498 (0.650 to 3.454)	6.471 (2.385 to 17.561) *	1.171 (0.474 to 2.892)	2.335 (0.953 to 5.721)	1.411 (0.603 to 3.305)	7.441 (2.919 to 18.969) *	1.094 (0.440 to 2.720)
Bronchopulmonary Dysplasia								
*OR (95%CI)*	4.358 (1.386 to 13.702) *	1.744 (0.594 to 5.117)	3.121 (0.951 to 10.241)	10.698 (1.350 to 84.789 *	2.188 (0.661 to 7.240)	1.646 (0.538 to 5.037)	13.270 (3.235 to 54.441) *	9.937 (1.240 to 79.634) *
Intraventricular Hemorrhage								
*OR (95%CI)*	3.184 (1.170 to 8.666) *	1.223 (0.488 to 3.065)	7.182 (2.219 to 23.244) *	0.518 (0.204 to 1.316)	3.850 (1.427 to 10.390) *	1.138 (0.457 to 2.834)	4.214 (1.591 to 11.162) *	0.498 (0.197 to 1.258)
Retinopathy of Prematurity								
*OR (95%CI)*	4.083 (1.339 to 12.447) *	1.028 (0.387 to 2.729)	12.174 (2.621 to 56.552) *	1.077 (0.379 to 3.057)	4.594 (1.496 to 14.102) *	0.927 (0.349 to 2.463)	6.178 (2.016 to 18.936) *	0.977 (0.347 to 2.749)
Hypotension								
*OR (95%CI)*	4.834 (2.047 to 11.418) *	0.793 (0.357 to 1.760)	7.563 (2.874 to 19.899) *	1.455 (0.607 to 3.490)	5.723 (2.446 to 13.393) *	0.732 (0.334 to 1.604)	3.798 (1.676 to 8.605) *	1.373 (0.589 to 3.204)
Morbidity°								
*OR (95%CI)*	4.058 (2.145 to 7.677) *	1.072 (0.581 to 1.979)	5.227 (2.814 to 9.708) *	0.974 (0.502 to 1.890)	4.109 (2.160 to 7.819) *	1.023 (0.548 to 1.912)	6.596 (3.441 to 12.643) *	0.986 (0.507 to 1.919)
Mortality								
*OR (95%CI)*	1.307 (0.414 to 4.126)	1.205 (0.396 to 3.664)	26.178 (3.210 to 213.479) *	0.475 (0.154 to 1.468)	1.519 (0.470 to 4.908)	1.148 (0.373 to 3.540)	10.977 (2.787 to 43.230) *	0.473 (0.153 to 1.460)

*hs-PDA* hemodynamically significant Patent Doctus Arteriosus

(^a^) Intramuscular steroid cycle in two doses of 12 mg over a 24-h period; ° BPD or NEC or IVH or PLV or ROP or Prolonged mechanical ventilation or Hypotension requiring treatment; * *p* < 0.05

When we analyzed Sub-group of children with GA ≤ 28 weeks and BW ≤ 1000 g, we found similar baseline characteristics, between Cases and Controls, but an increased rate of mother with pregnancy-induced hypertension and occurrence of BPD in those with hs-PDA compared with Controls (Tables 1 and 2). The mortality rate was similar between Cases (19.5%) and Controls (27.8%) in this sensitivity analysis. In this Sub-Group of patients, we observed that the mean length of hospital stay was significantly increased in Cases (98.8, 95%CI 79.0 to 118.7) compared with Controls (64.4, 95%CI 44.6 to 84.2, *p =* 0.034).

In a sensitivity binary logistic regression analysis, including newborns of Sub-groups A and B, hs-PDA was a significant and independent factor associated with occurrence of BPD (Table 4).

**Table 4:** Binary logistic regression analysis evaluating influence of covariates on morbidity and mortality in study population with gestational age ≤ 28 weeks and birth weight ≤ 1000 g

*Dependent variabels*	Covariates		
	hs-PDA	Gender	Pregnancy-induced hypertension
Prolonged Mechanical Ventilation			
*OR (95%CI)*	3.097 (0.731 to 13.132)	1.210 (0.391 to 3.750)	1.415 (0.398 to 5.033)
Bronchopulmonary Dysplasia			
*OR (95%CI)*	8.489 (0.968 to 74.435) *	2.112 (0.572 to 7.800)	0.689 (0.166 to 2.859)
Intraventricular Hemorrhage			
*OR (95%CI)*	1.466 (0.365 to 5.894)	2.870 (0.834 to 9.878)	0.713 (0.173 to 2.946)
Retinopathy of Prematurity			
*OR (95%CI)*	3.553 (0.674 to 18.742)	0.865 (0.260 to 2.879)	1.583 (0.424 to 5.903)
Hypotension			
*OR (95%CI)*	2.405 (0.669 to 8.649)	0.823 (0.278 to 2.440)	0.520 (0.141 to 1.924)
Morbidity°			
*OR (95%CI)*	2.671 (0.674 to 10.592)	1.248 (0.349 to 4.462)	0.853 (0.176 to 4.139)
Mortality			
*OR (95%CI)*	0.899 (0.211 to 3.830)	2.212 (0.548 to 8.932)	0.209 (0.023 to 1.915)

*hs-PDA* hemodynamically significant Patent Doctus Arteriosus. (^a^) Intramuscular steroid cycle in two doses of 12 mg over a 24-h period; ° BPD or NEC or IVH or PLV or ROP or Prolonged mechanical ventilation or Hypothension; * *p* < 0.05

We observed, in a sensitivity linear logistic regression analysis, that hs-PDA was not an independent and significant risk factor for length of hospital stay (Table S1).

## Discussion

In our population of newborns, with GA < 32 weeks or BW < 1500 g, occurrence of the main morbidities of prematurity seems to be related to hs-PDA. However, this observed association depended also on GA, BW, and use of prenatal steroids. When we corrected data for confounding variables, we confirmed that hs-PDA represents a risk factor for the occurrence of prolonged mechanical ventilation, BPD, IVH, ROP, and hypotension, but along with GA, BW and prenatal use of steroids. In other words, in keeping with our findings, it is not possible to exclude that morbidity depends on factors other than hs-PDA.

We observed a different scenario in extremely premature babies (GA ≤ 28 weeks and BW ≤ 1000 g). In particular, hs-PDA resulted as an independent risk factor for BPD, which is one of the most important morbidity of prematurity, in newborns with GA ≤ 28 weeks and BW ≤ 1000 g, even if we corrected data for GA.

Finally, we showed that the presence of hs-PDA may increase the duration of hospitalization in enrolled newborns.

It has been hypothesized that hemodynamic condition generated by hs-PDA may induce different organs injuries, leading to an increased risk of clinical complications [24]. Despite it is clinical plausible, there is still uncertainty from current evidence regarding possible association between hs-PDA, morbidity and mortality in VLBW infants. Randomized controlled trials (RCTs) did not clarify whether PDA may have clinical consequences in this population, whereas, several not controlled studies found a relation between symptomatic hs-PDA, morbidity and mortality [25–28]. A retrospective study, exploring the impact of PDA on survival, found that mortality rate was higher among preterm infants with PDA [2]. In those studies, it is not possible to determine if the adverse neonatal outcomes observed in preterm newborns can be referred to hs-PDA or to GA.

In this study, all patients enrolled had a hs-PDA and confidence intervals for the odds for mortality were relatively wide. We found that hs-PDA influenced morbidity in a more complex regression model, but solely in association with other variables. Contemporarily, we observed that hs-PDA did not independently affect survival, neither in VLBW nor in extremely low gestational age neonates (ELGAN) and ELBW infants. In addition, that the presence of a hs-PDA could, in turn, depend on GA and BW. Thus, the clinical validity of the multivariate model, used in this study, should take into account this aspect.

Our results suggest that the presence of hs-PDA may prolong the duration of mechanical ventilation. A persistent shunt, increasing the pulmonary blood flow, can lead to pulmonary edema, loss of lung compliance, and deterioration of respiratory status. In a retrospective cohort study was assessed the relation between PDA and prolonged mechanical ventilation in preterm newborns [25, 29]. Although a causal association has not been proven, the link was greatest among infants born at lower gestational age who were treated with mechanical ventilation in the presence of a large ductal shunt [29]. In addition, authors did not perform a multivariate analysis in order to confirm their findings adjusted for other covariates. We did not found any association between hs-PDA and occurrence of prolonged mechanical ventilation in ELGAN and ELBW enrolled in our study, neither in univariate analysis nor in multivariate analysis. However, the difference in the rate of patients with prolonged mechanical ventilation, observed between Cases and Controls, might become significant in a larger population of ELGAN and ELBW newborns.

Prolonged mechanical ventilation could be also a condition that may have increased the risk of BPD in enrolled newborns affected by hs-PDA. It has been well described by Willis et al. that prolonged mechanical ventilation in preterm newborns can determine alveolar simplification leading to increased likely to develop BPD [30]. Epidemiological studies demonstrated a strong association between the presence of a PDA and the development of BPD [30].

A causative relation between the PDA and the development of BPD was suggested by observational retrospective evidence [28, 31]. Schena et al. estimated that occurrence of BPD depended on severity of hs-PDA exposure. El-Khuffash et al. in a study exploring possible prognostic values of a PDA score based on echocardiography measurements found that association between PDA and BPD strengthens with decreasing gestational age [24]. On the other hand, in a recent study comparing mandatory closure vs. a non-interventional approach to manage hs- PDA in preterm newborns, despite longer PDA exposure, the non-interventional approach was associated with significantly less BPD [32]. However, all newborns with hs-PDA enrolled in this study required ventilatory treatment, which could have influenced the occurrence of BPD. We observed a relation between hs-PDA and BPD still when we corrected the data for confounding variables including GA and BW. This association was observed in a multivariate analysis performed in the Sub-group of ELGAN and ELBW subjects. However, the significance of multivariate model suggests that further studies, including a larger number of preterm newborns, should be performed to confirm these findings.

We observed a relation between hs-PDA and IVH a suggested by previous retrospective studies. A large retrospective cohort study on VLBW infants demonstrated that the rate of spontaneous closure before hospital discharge occurs in the majority of patients. However, the Authors excluded the deceased infants from the analysis, whose cause of death was reported to be potentially related to PDA, including IVH and all of them had documented PDA [33]. Our multivariate analysis showed that hs-PDA was a risk factor in association with GA and BW for IVH. Further studies are needed to establish the causality between hemodynamic consequences of PDA and occurrence of IVH.

We showed the association between PDA and ROP, as observed in previous studies. Results from recent wide prospective study showed that PDA requiring medication significantly correlated with the development of ROP. This research, as well as our study, found that PDA was significantly correlated with ROP in the multivariate logistic regression analysis adjusted for GA and BW [34]. It is known that pO_2_ regulates the production of predominant regulators of retinal angiogenesis, therefore O_2_ therapy could be responsible for the retinal damage associated with ROP pathogenesis [35]. However, taking these results into account, it is possible to hypothesize that hs-PDA, in critical ill newborns, may contribute to increase the risk of ROP due to hemodynamic instability that, in turn, may influence development of the immature and incompletely vascularized retina [36]. We did not observe the association between hs-PDA and ROP in ELGAN and ELBW patients. Probably, in these subjects, the extremely immaturity of retina vascularization represents a major risk factor for ROP independently by the presence of hs-PDA.

We observed a significant increased risk of hypotension in newborns with hs-PDA. Our findings are in keeping with the results of Ratner et al. who showed that a hs-PDA was associated with a reduction in systolic and diastolic blood pressure [37]. Although it is true that a moderate-to-large PDA can lower systemic blood pressure and is associated with the presence of hypotension at the end of the first week [14, 38, 39], no studies to date has determined whether the PDA actually is responsible for the hypotension or whether its presence is just a surrogate for nonspecific causes related to immaturity/illness. Our multivariate analysis suggested that hypotension in preterm newborns depend contemporarily on hs-PDA, BW and GA. However, the hs-PDA does not seem to influence the occurrence of hypotension in ELGAN and ELBW subjects. Clinical validity of these results should be confirmed in a specifically designed clinical trial focused on hypotension.

A causal relationship between pregnancy-induced hypertension and occurrence of PDA has been described in previous studies. According with a recent Cohort research, we demonstrated a higher rate of pregnancy-induced hypertension in Cases compared with Controls. That increased risk for developing PDA in newborns of women with preeclampsia has been attributed to the likelihood of shared angiogenic imbalance between the mother and fetus [40–42]. However, this relation did not persist in our multivariate analysis.

A large number of previous piece of research have included infants with PDA regardless of hemodynamic significance [43–45]. Therefore, any effect of PDA may have been masked by the absence of clinical consequences of a physiological condition.

Our results should be interpreted considering study limitations. The association between hs-PDA and morbidity outcome in VLBW may be related to the effects of chance, random error, bias or confounding factors. We verified that effect on the main outcome of the study persisted even after correction for confounding variables. Despite of everything, other confounding variables, unknown or not considered in our statistical analysis, may have influenced the study results. In addition, covariate “hs-PDA” could dependent by GA and BW.

We designed a Case-Control study. We observed difference in the baseline clinical characteristics between the two Groups of newborns enrolled in the study. To overcome this limit, we performed a multivariate analysis. However, in this analysis, hs-PDA represent a risk factor for morbidity only in association with other confounding variables such as GA, BW and use of prenatal steroids. Despite of everything, other confounding variables, unknown or not considered in our statistical analysis, may have influenced the study results. We also performed a Subgroup analysis for a population of newborns with GA ≤ 28 weeks and BW ≤ 1000 g. In this way, we analyzed a Sub population with similar GA, overcoming the limitations of its retrospective design. However, the small number of patience included in this sub-analysis limit the generalization of results. Further studies are necessary to better address this issue.

The ECHO data include only such variables useful for assessing the hemodynamic significance of a PDA. Despite other parameters (e.g. left ventricular output, pulmonary vein flowmetry and isovolemic relaxation time) could be useful to define hemodynamic role of PDA, these informations are not registered in the clinical charts of the patience, and thus are not available for our retrospective study. Assessing PDA significance by ECHO, is labor-intensive, requires significant skill and is subject to considerable operator variability. In our policy, we preferred to use ECHO parameters for which our cardiologists had high expertise. However, there is a lack of accuracy for a standard definition of hs-PDA. Thus, different diagnostic assessments could have led to different conclusions. To limit observer bias, researchers unaware for study aims collected the data for the analysis, a third party observer was involved to collect data on the outcome of the study, and a blinded statistician performed the data analysis. Finally, Results regarding population of ELGAN and ELBW newborns should be interpreted considering the small number of subjects enrolled as Controls compared with Cases.

## Conclusion

In conclusion, the presence of hs-PDA does not seem to influence the clinical course of VLBW newborns, but only that of ELGAN and ELBW newborns. Potential association between hs-PDA and BPD, found in our case-control retrospective study, encourages designing a randomized trial, that should include an adequate number of critical newborns, with GA ≤ 28 weeks and BW ≤ 1000 g.

## Supplementary Information

**Additional file 1.**

## Abbreviations

PDA: Patent ductus arteriosus
ELBW: Extremely low birth weight
hs-PDA: Hemodynamic significant patent ductus arteriosus
VLBW: Very low birth weight
GA: Gestational age
BW: Birth weight
NICU: Neonatal intensive care unit
ECHO: Echocardiography
BPD: Bronchopulmonary dysplasia
IVH: Intraventricular hemorrhage
NEC: Necrotizing enterocolitis
ROP: Retinopathy of prematurity
RCTs: Randomized clinical trials
ELGAN: Extremely low gestational age
