# Supplementary material for: Morbidity associated with patent ductus arteriosus in preterm newborns: a retrospective case-control study
Source: Ital J Pediatr. 2021 Jan 14;47:9. doi: 10.1186/s13052-021-00956-2 (PMC7809822; doi:10.1186/s13052-021-00956-2)
Supplement: Supplementary file 1 — Additional file 1. [file 13052_2021_956_MOESM1_ESM.docx]

**Supplementary Table**

**Table S1. Linear regression analysis evaluating influence of covariates on secondary outcome.**

| *Covariates* | | | B | Std. Err. | β |
| --- | --- | --- | --- | --- | --- |
| **Overall** | Model 1 | *Gender* | 0.873 | 4.111 | 0.012 |
|  |  | *GA* | -5.259 * | 0.888 | -0.368 |
|  |  | *Antenatal steroids^a^* | 11.497 * | 4.507 | 0.144 |
|  |  | *hs-PDA* | 12.062 * | 5.020 | 0.150 |
|  | Model 2 | *Gender* | 2.329 | 4.046 | 0.032 |
|  |  | *BW* | -0.040 * | 0.006 | -0.394 |
|  |  | *Antenatal steroids^a^* | 12.440 * | 4.430 | 0.156 |
|  |  | *hs-PDA* | 14.508 * | 4.689 | 0.181 |
| **Gestational Age ≤ 28 weeks and Birth Weight**  **≤ 1000 grams** | Model 1 | *Gender* | 1.006 | 14.949 | 0.009 |
|  |  | *GA* | -1.895 | 4.981 | -0053 |
|  |  | *Pregnancy-induced hypertension* | 31.294 | 18.823 | 0.239 |
|  |  | *hs-PDA* | 25.172 | 16.710 | 0.209 |
|  | Model 2 | *Gender* | 1.708 | 14.808 | 0.015 |
|  |  | *BW* | 0.021 | 0.052 | 0.054 |
|  |  | *Pregnancy-induced hypertension* | 29.976 | 18.184 | 0.229 |
|  |  | *hs-PDA* | 26.404 | 16.595 | 0.219 |

**Notes.** GA: Gestational Age; hs-PDA: hemodynamically significant Patent Doctus Arteriosus; BW: Birth weight; (a) Intramuscular steroid cycle in two doses of 12 mg over a 24-hour period; * *p* < 0.05.
